# A new approach for estimating VAR systems in the mixed-frequency case

**DOI:** 10.1007/s00362-018-0985-1

**Published:** 2018-02-03

**Authors:** Lukas Koelbl, Manfred Deistler

**Affiliations:** 1 Accenture Digital, Accenture Austria, Vienna, Austria; 2 Institute of Statistics and Mathematical Methods in Economics, Vienna University of Technology, Vienna, Austria

**Keywords:** High-frequency VAR, Mixed-frequency data, Estimation procedure

## Abstract

In this paper we present a new estimation procedure named MF-IVL for VAR systems in the case of mixed-frequency data, where the data maybe, e.g., stock or flow data. The main idea of this new procedure is to project the slow components on the present and past fast ones in order to create instrumental variables. This procedure is shown to be generically consistent. Our claim is that the procedure is fast and more accurate when compared to the extended Yule-Walker procedure. A comparison of these two procedures is given by simulation.

## Introduction

We propose a simple and fast algorithm for estimating the parameters in a multivariate high-frequency VAR system from mixed-frequency data. The VAR system is of the form

1.1 $$\begin{aligned} y_{t}= \begin{pmatrix} y_{t}^{f}\\ y_{t}^{s} \end{pmatrix} =A_{1}y_{t-1}+\dots +A_{p}y_{t-p}+\nu _{t},\,t\in {\mathbb {Z}}, \end{aligned}$$

where $A_{i}\in {\mathbb {R}}^{n\times n}$ and the AR order *p* is given. Throughout we assume the stability condition

1.2 $$\begin{aligned} \det \left( a(z)\right) \ne 0\,\left| z\right| \le 1, \end{aligned}$$

where $a(z)=I_{n}-A_{1}z-\dots -A_{p}z^{p}$. Here *z* is used for the complex variable as well as for the backward shift on the integers ${\mathbb {Z}}$. We assume that $\left( \nu _{t}\right)$ is white noise and we only consider the stable steady state solution $y_{t}=a(z)^{-1}\nu _{t}$. The innovation covariance matrix

1.3 $$\begin{aligned} \Sigma _{\nu }={\mathbb {E}}\left( \nu _{t}\nu _{t}^{T}\right) >0 \end{aligned}$$

is assumed to be non-singular. The parameter space for the high-frequency models considered is:

1.4 $$\begin{aligned} \Theta \!=\!\left\{ \left( A_{1},\dots ,A_{p}\right) \left| \right. \det \left( a(z)\right) \ne 0,\,\left| z\right| \le 1\right\} \times \left\{ \text {vech}\left( \Sigma _{\nu }\right) \left| \right. \Sigma _{\nu }\!=\Sigma _{\nu }^{T},\Sigma _{\nu }>0\right\} \! ,\nonumber \\ \end{aligned}$$

where $\text {vech}$ is the half-vectorization. Note that the conditions (1.2) and (1.3) define an open subset in the euclidean space ${\mathbb {R}}^{pn^{2}+n\left( n+1\right) /2}$. A subset of $\Theta$ is called generic if it contains an open and dense subset of $\Theta$. The VAR system (1.1) can be written in state space form as

1.5 $$\begin{aligned} \underbrace{\begin{pmatrix}y_{t}\\ y_{t-1}\\ \vdots \\ y_{t-p+1} \end{pmatrix}}_{x_{t+1}}&=\underbrace{\begin{pmatrix}A_{1} &{} \cdots &{} A_{p-1} &{} A_{p}\\ I_{n}\\ &{} \ddots \\ &{} &{} I_{n} &{} 0 \end{pmatrix}}_{{\mathcal {A}}}\underbrace{\begin{pmatrix}y_{t-1}\\ y_{t-2}\\ \vdots \\ y_{t-p} \end{pmatrix}}_{x_{t}}+\underbrace{\begin{pmatrix}I_{n}\\ 0\\ \vdots \\ 0 \end{pmatrix}}_{{\mathcal {B}}}\nu _{t}, \end{aligned}$$

1.6 $$\begin{aligned} y_{t}&=\begin{pmatrix}A_{1}&\cdots&A_{p}\end{pmatrix}x_{t}+\nu _{t}. \end{aligned}$$

In this paper we consider the problem of estimating the parameters of the *n*-dimensional high-frequency VAR model (1.1) using mixed-frequency data. We actually observe mixed-frequency data of the form

1.7 $$\begin{aligned} \begin{pmatrix}y_{t}^{f}\\ w_{t} \end{pmatrix}, \end{aligned}$$

where

1.8 $$\begin{aligned} w_{t}=\sum _{i=1}^{N}c_{i}y_{t-i+1}^{s}, \end{aligned}$$

where $c_{i}\in {\mathbb {R}}$, $1<N\in {\mathbb {N}}$ and at least one $c_{i}\ne 0$. Here the $n_{f}$-dimensional, say, fast component $y_{t}^{f}$ is observed at the highest (sampling) frequency $t\in {\mathbb {Z}}$ and the $n_{s}$-dimensional slow component $w_{t}$ is observed only for $t\in N{\mathbb {Z}}$, i.e. for every *N*-th time point. In this paper we assume that $n_{f}\ge 1$. The population second moments, which can be directly observed, are of the form

1.9 $$\begin{aligned} \gamma ^{ff}(h)= & {} {\mathbb {E}}\left( y_{t+h}^{f}\left( y_{t}^{f}\right) ^{T}\right) ,\quad h\in {\mathbb {Z}},\nonumber \\ \gamma ^{wf}(h)= & {} {\mathbb {E}}\left( w_{t+h}\left( y_{t}^{f}\right) ^{T}\right) ,\quad h\in {\mathbb {Z}},\nonumber \\ \gamma ^{ww}(h)= & {} {\mathbb {E}}\left( w_{t+h}w_{t}^{T}\right) ,\quad h\in N{\mathbb {Z}}. \end{aligned}$$

Generic identifiability of the high-frequency parameters $A_{i}$, $i=1,\ldots ,p$ and $\Sigma _{\nu }$ has been shown in Anderson et al. (2016) (Theorems 2 and 3). Estimation procedures, in particular, a procedure based on the extended Yule-Walker (XYW) equations [see Chen and Zadrozny (1998)] and a procedure based on the Gaussian Likelihood as well as an EM algorithm are discussed in Koelbl et al. (2016) and Koelbl (2015). There it is shown that the MLE as well as the EM estimator heavily depend on the initial estimator used. The purpose of this paper is to describe an estimation procedure which can be used as an initial estimator, e.g. for the EM algorithm, but also as an estimator on its own, because it is easy to calculate, consistent and outperforms the estimator based on the XYW equations.

## The mixed-frequency IVL estimator

### The stock case

For the case of stock variables (i.e. $c_{1}=1$, $c_{i}=0$, $i=2,\ldots ,N$) the second moments, which can be directly observed, are:

2.1 $$\begin{aligned} \gamma ^{ff}(h)= & {} {\mathbb {E}}\left( y_{t+h}^{f}\left( y_{t}^{f}\right) ^{T}\right) ,\quad h\in {\mathbb {Z}},\nonumber \\ \gamma ^{sf}(h)= & {} {\mathbb {E}}\left( y_{t+h}^{s}\left( y_{t}^{f}\right) ^{T}\right) ,\quad h\in {\mathbb {Z}},\nonumber \\ \gamma ^{ss}(h)= & {} {\mathbb {E}}\left( y_{t+h}^{s}\left( y_{t}^{s}\right) ^{T}\right) ,\quad h\in N{\mathbb {Z}}. \end{aligned}$$

In Anderson et al. (2016) it is shown that the system parameters can be generically reconstructed with the help of the extended Yule-Walker (XYW) equations, which can be constructed by postmultiplying Eq. (1.1) by $\left( y_{t-j}^{f}\right) ^{T},\ j=1,\ldots ,np$ and forming expectations:

$$\begin{aligned}&{\mathbb {E}}\underbrace{\left[ y_{t}\left( \left( y_{t-1}^{f}\right) ^{T},\dots ,\left( y_{t-np}^{f}\right) ^{T}\right) \right] }_{=:Z_{1}}\\&\quad =(A_{1},\dots ,A_{p})\underbrace{{\mathbb {E}} \left[ \begin{pmatrix}y_{t-1}\\ \vdots \\ y_{t-p} \end{pmatrix}\left( \left( y_{t-1}^{f}\right) ^{T},\dots ,\left( y_{t-np}^{f}\right) ^{T}\right) \right] }_{=:Z_{0}}. \end{aligned}$$

Note, that the second moments on the left as well as those on the right hand side of the above equation can be directly observed in the mixed-frequency stock case. In Anderson et al. (2016) Theorem 2, it is shown that $Z_{0}$ has generically full row rank and therefore we generically obtain $(A_{1},\dots ,A_{p})=Z_{1}Z_{0}^{T}\left( Z_{0}Z_{0}^{T}\right) ^{-1}$. The XYW estimators are obtained by replacing the population second moments by their sample counterparts:

2.2 $$\begin{aligned} {\hat{\gamma }}^{ff}(h)= & {} \frac{1}{T}\sum _{t=1}^{T-h}y_{t+h}^{f}\left( y_{t}^{f}\right) ^{T},\quad h\ge 0, \end{aligned}$$

2.3 $$\begin{aligned} {\hat{\gamma }}^{ff}(h)= & {} {\hat{\gamma }}^{ff}(-h)^{T}, \end{aligned}$$

2.4 $$\begin{aligned} {\hat{\gamma }}^{sf}(h)= & {} \frac{1}{T/N}\sum _{t}y_{Nt}^{s}\left( y_{Nt-h}^{f}\right) ^{T}, \end{aligned}$$

where the estimator of $\gamma ^{sf}(h)$ has only (approximately) ${1}/{N}$-th of the summands compared to the estimator of $\gamma ^{ff}(h)$ due to the missing observations [see Koelbl et al. (2016)].

The new estimation procedure proposed is as follows: The basic idea is to generate instrumental variables by projecting the slow components $y_{t}^{s}$ on the space generated by present and a sufficient number of lagged fast components $y_{j}^{f}$. To be more precise, let, for a suitable chosen $1\le k\le t$, ${\mathcal {H}}_{k}^{f}(t)=\text {span}\left\{ y_{j}^{f}:\,t-k\le j\le t\right\}$ be the Hilbert space spanned by the one-dimensional components of the $y_{j}^{f}$ in the underlying space of square integrable random variables ${\mathcal {L}}^{2}$ over $\left( \Omega ,{\mathcal {A}},P\right)$ and let $x_{t|t-1}^{k}$ denote the (componentwise) projection of the state $x_{t}$ onto ${\mathcal {H}}_{k}^{f}(t-1)$ written as $x_{t|t-1}^{k}={\mathbb {P}}_{{\mathcal {H}}_{k}^{f}(t-1)}\left( x_{t}\right)$. Projecting the state Eq. (1.5) onto ${\mathcal {H}}_{k}^{f}(t)$, we obtain, using an obvious notation,

2.5 $$\begin{aligned} x_{t+1|t}^{k}={\mathcal {A}}x_{t|t-1}^{k}+\left\{ {\mathcal {A}}\left( x_{t|t}^{k}-x_{t|t-1}^{k}\right) +{\mathcal {B}}\nu _{t|t}^{k}\right\} . \end{aligned}$$

In a first step we show that the matrix ${\mathbb {E}}\left( x_{t|t-1}^{k}\left( x_{t|t-1}^{k}\right) ^{T}\right)$ is generically non-singular for $k\ge np-1$: For $k_{0}=np-1$ and $$Y_{t,k}^{-}=\begin{pmatrix}\left( y_{t}^{f}\right) ^{T},\left( y_{t-1}^{f}\right) ^{T},\ldots ,\left( y_{t-k}^{f}\right) ^{T}\end{pmatrix}^{T}$$ let $\Gamma ^{ff}\left( k\right) ={\mathbb {E}}\left( Y_{t,k}^{-}\left( Y_{t,k}^{-}\right) ^{T}\right)$. It follows that $\Gamma ^{ff}\left( k_{0}\right) >0$ which is a direct consequence of $\Sigma _{\nu }>0$. The projection $x_{t|t-1}^{k_{0}}$ is obtained by using the OLS formula

$$\begin{aligned} x_{t|t-1}^{k_{0}}=\underbrace{{\mathbb {E}}\left( x_{t}\left( Y_{t-1,k_{0}}^{-}\right) ^{T}\right) }_{Z_{0}}\Gamma ^{ff}\left( k_{0}\right) ^{-1}Y_{t-1,k_{0}}^{-} \end{aligned}$$

and therefore generically

2.6 $$\begin{aligned} {\mathbb {E}}\left( x_{t|t-1}^{k_{0}}\left( x_{t|t-1}^{k_{0}}\right) ^{T}\right) =Z_{0}\Gamma ^{ff}\left( k_{0}\right) ^{-1}Z_{0}^{T}>0 \end{aligned}$$

since $Z_{0}$ has generically full row rank. This implies that generically

2.7 $$\begin{aligned} {\mathcal {A}}=\left( {\mathbb {E}}x_{t+1|t}^{k}\left( x_{t|t-1}^{k}\right) ^{T}\right) \left( {\mathbb {E}}x_{t|t-1}^{k}\left( x_{t|t-1}^{k}\right) ^{T}\right) ^{-1} \end{aligned}$$

holds, since $x_{t|t-1}^{k}$ is uncorrelated with $\left( x_{t|t}^{k}-x_{t|t-1}^{k}\right)$ and $\nu _{t|t}^{k}$. Note that, for $k\ge p-1$,

$$\begin{aligned} x_{t|t-1}^{k}=\begin{pmatrix}y_{t-1}^{f}\\ {\mathbb {P}}_{{\mathcal {H}}_{k}^{f}(t-1)}\left( y_{t-1}^{s}\right) \\ \vdots \\ y_{t-p}^{f}\\ {\mathbb {P}}_{{\mathcal {H}}_{k}^{f}(t-1)}\left( y_{t-p}^{s}\right) \end{pmatrix}. \end{aligned}$$

An estimator of the state $x_{t+1|t}^{k}$, denoted by ${\hat{x}}_{t+1|t}^{k}$, can be constructed as follows: W.l.o.g. let $p=2$ and $N=2$. The first *n* components of ${\hat{x}}_{t+1|t}^{k}$ can be estimated by projecting $y_{t}$ onto $y_{t}^{f},\dots ,y_{t-k}^{f}$. This can be done by estimating $\beta _{1}$ in

2.8 $$\begin{aligned} y_{t}=\beta _{1}Y_{t,k}^{-}+\varepsilon _{t},\quad t\in 2{\mathbb {Z}}, \end{aligned}$$

Let ${\hat{\beta }}_{1,T}$ denote the OLS estimator of $\beta _{1}$. Then we obtain $\left( I_{n},0\right) {\hat{x}}_{t+1|t}^{k}={\hat{\beta }}_{1,T}Y_{t,k}^{-}$, $t\in {\mathbb {Z}}$. The second *n* components of $x_{t+1|t}^{k}$ must be, due to the mixed-frequency structure and $N=2$, estimated in a different way: Analogously to (2.8) we can construct

2.9 $$\begin{aligned} y_{t-1}=\beta _{2}Y_{t,k}^{-}+\zeta _{t}, \end{aligned}$$

but now we cannot directly observe the left hand side of (2.9). Therefore, we must shift (2.9) to

2.10 $$\begin{aligned} y_{t}=\beta _{2}Y_{t+1,k}^{-}+\zeta _{t+1},\quad t\in 2{\mathbb {Z}} \end{aligned}$$

which directly leads us to the OLS estimator ${\hat{\beta }}_{2,T}$ of $\beta _{2}$, since the left as well as the right hand side of (2.10) can be directly observed. In a last step we can construct the remaining part of the state with the help of $\left( 0,I_{n}\right) {\hat{x}}_{t+1|t}^{k}={\hat{\beta }}_{2,T}Y_{t,k}^{-}$, $t\in {\mathbb {Z}}$, which leads us to

2.11 $$\begin{aligned} {\hat{x}}_{t+1|t}^{k}= \begin{pmatrix}{\hat{\beta }}_{1,T}\\ {\hat{\beta }}_{2,T} \end{pmatrix}Y_{t,k}^{-},\quad t\in {\mathbb {Z}}. \end{aligned}$$

Using these instrumental variables, we can estimate ${\mathcal {A}}$ according to (2.7):

2.12 $$\begin{aligned} \hat{{\mathcal {A}}}_{T}=\left( \sum _{t=p+1}^{T-1}{\hat{x}}_{t+1|t}^{k}\left( {\hat{x}}_{t|t-1}^{k}\right) ^{T}\right) \left( \sum _{t=p+1}^{T}{\hat{x}}_{t|t-1}^{k}\left( {\hat{x}}_{t|t-1}^{k}\right) ^{T}\right) ^{-1}. \end{aligned}$$

#### Theorem 1

Under the additional assumptions, that $\underset{T\rightarrow \infty }{\lim }\frac{1}{T}\sum _{t=1}^{T}\nu _{t}\nu _{t}^{T}=\Sigma _{\nu }$ a.s., we have $\underset{T\rightarrow \infty }{\lim }\hat{{\mathcal {A}}}_{T}={\mathcal {A}}$ a.s.

#### Proof

Again we assume that $p=2$ and $N=2$. The above condition $\underset{T\rightarrow \infty }{\lim }\frac{1}{T}\sum _{t=1}^{T}\nu _{t}\nu _{t}^{T}=\Sigma _{\nu }$ implies, see Hannan and Deistler (2012) Theorem 4.1.1, that

2.13 $$\begin{aligned} \underset{T\rightarrow \infty }{\lim }\frac{1}{T}\sum _{t=1}^{T-h}y_{t+h}y_{t}^{T}=\gamma \left( h\right) , \end{aligned}$$

where $\gamma \left( j\right) ={\mathbb {E}}\left( y_{t}y_{t-j}^{T}\right)$. In a next step, let us write (2.6) as

2.14 $$\begin{aligned} {\mathbb {E}}\left( x_{t|t-1}^{k_{0}}\left( x_{t|t-1}^{k_{0}}\right) ^{T}\right)&=Z_{0}\Gamma ^{ff}\left( k_{0}\right) ^{-1}\Gamma ^{ff}\left( k_{0}\right) \Gamma ^{ff}\left( k_{0}\right) ^{-1}Z_{0}^{T}\\&=\begin{pmatrix}\beta _{1}\\ \beta _{2} \end{pmatrix}\Gamma ^{ff}\left( k_{0}\right) \begin{pmatrix}\beta _{1}\\ \beta _{2} \end{pmatrix}^{T}\nonumber \end{aligned}$$

Equation (2.13) implies that ${\hat{\beta }}_{1,T}$ and ${\hat{\beta }}_{2,T}$ are consistent estimators for $\beta _{1}$ and $\beta _{2}$, respectively. Thus, we obtain that

$$\begin{aligned} \underset{T\rightarrow \infty }{\lim }\frac{1}{T}\sum _{t=k+2}^{T+1}{\hat{x}}_{t|t-1}^{k_{0}}\left( {\hat{x}}_{t|t-1}^{k_{0}}\right) ^{T}&=\underset{T\rightarrow \infty }{\lim }\frac{1}{T}\sum _{t=k+2}^{T+1} \begin{pmatrix}{\hat{\beta }}_{1,T}\\ {\hat{\beta }}_{2,T} \end{pmatrix}Y_{t-1,k}^{-}\left( Y_{t-1,k}^{-}\right) ^{T} \begin{pmatrix}{\hat{\beta }}_{1,T}\\ {\hat{\beta }}_{2,T} \end{pmatrix}^{T}\\&=\underset{T\rightarrow \infty }{\lim } \begin{pmatrix}{\hat{\beta }}_{1,T}\\ {\hat{\beta }}_{2,T} \end{pmatrix}{\hat{\Gamma }}^{ff}\left( k_{0}\right) \begin{pmatrix}{\hat{\beta }}_{1,T}\\ {\hat{\beta }}_{2,T} \end{pmatrix}^{T}\\&={\mathbb {E}}\left( x_{t|t-1}^{k_{0}}\left( x_{t|t-1}^{k_{0}}\right) ^{T}\right) . \end{aligned}$$

An analogous result can be shown for ${\mathbb {E}}\left( x_{t+1|t}^{k}\left( x_{t|t-1}^{k}\right) ^{T}\right)$. This concludes the proof. $\square$

Of course the choice of *k* is important for estimating the system parameters. Our approach is to regress $y_{t}^{s}$ on $y_{t}^{f},\dots ,y_{t-k}^{f}$ and to determine the maximum lag *k* by using AIC. Note that the structure of the matrix ${\mathcal {A}}$, as far as the a priori zeros and ones are concerned, is not preserved by the estimation procedure (2.12). For this reason, we define a new estimator for the system parameters as

2.15 $$\begin{aligned} \hat{\hat{\mathcal {A}}}_{T}= \begin{pmatrix} \left( I_{n},0,\ldots ,0\right) \hat{{\mathcal {A}}}\\ \left( \begin{array}{cc} I_{n(p-1)}, &{} 0 \end{array}\right) \end{pmatrix}. \end{aligned}$$

Clearly, $\hat{\hat{\mathcal {A}}}_{T}$ is also consistent. As shown in Anderson et al. (2016), the innovation covariance matrix $\Sigma _{\nu }$ can be generically consistently estimated according to the following formula

2.16 $$\begin{aligned} \text {vec}\left( \Sigma _{\nu }\right) =\left( ({\mathcal {G}}\otimes {\mathcal {G}})\left( I_{(np)^{2}}-\left( {\mathcal {A}}\otimes {\mathcal {A}}\right) \right) ^{-1}({\mathcal {G}}^{T}\otimes {\mathcal {G}}^{T})\right) ^{-1}\text {vec}\left( \gamma (0)\right) \end{aligned}$$

where ${\mathcal {G}}=\left( I_{n},0,\ldots ,0\right)$ and where $\otimes$ denotes the Kronecker symbol. Let ${\hat{\Sigma }}_{\nu }$ denote the corresponding estimator.

Note that the estimator $\hat{\hat{\mathcal {A}}}_{T}$ (denoted by MF-IVL estimator) neither necessarily gives a stable AR system, nor is ${\hat{\Sigma }}_{\nu }$ necessarily positive definite. Projecting a symmetric matrix on the space of positive definite symmetric matrices is in a certain sense a standard procedure (see Higham 1989; Koelbl 2015). Projecting unstable system parameters on the space of stable ones is described in Koelbl (2015) and, for the univariate case, in Orbandexivry et al. (2013). Projecting slow variables on fast lagged variables is also mentioned in Ghysels et al. (2007).

### The flow case

For the case of the more general observation scheme (1.8), we proceed as follows: Let

2.17 $$\begin{aligned} z_{t}=\sum _{i=1}^{N}c_{i}y_{t-i+1}= \begin{pmatrix} \sum \nolimits _{i=1}^{N}c_{i}y_{t-i+1}^{f}\\ w_{t} \end{pmatrix}. \end{aligned}$$

From (1.5) we obtain

2.18 $$\begin{aligned} \underbrace{\begin{pmatrix}z_{t}\\ z_{t-1}\\ \vdots \\ z_{t-p+1} \end{pmatrix}}_{f_{t+1}}={\mathcal {A}} \underbrace{\begin{pmatrix}z_{t-1}\\ z_{t-2}\\ \vdots \\ z_{t-p} \end{pmatrix}}_{f_{t}}+{\mathcal {B}}\left( \sum _{i=1}^{N}c_{i}\nu _{t-i+1}\right) . \end{aligned}$$

Let $f_{t+1|t}^{k}$ denote the projection of $f_{t+1}$ on the space ${\mathcal {H}}_{k}^{f}\left( t\right)$. Projecting both sides of (2.18) on the space ${\mathcal {H}}_{k}^{f}\left( t\right)$ we get in an obvious notation

2.19 $$\begin{aligned} f_{t+1|t}^{k}={\mathcal {A}}f_{t|t-N}^{k}+\left\{ {\mathcal {A}}\left( f_{t|t}^{k}-f_{t|t-N}^{k}\right) +{\mathcal {B}}\left( \sum _{i=1}^{N}c_{i}\nu _{t-i+1|t}\right) \right\} . \end{aligned}$$

Post-multiplying (2.19) by $\left( f_{t|t-N}^{k}\right) ^{T}$ and taking the expectations we obtain

2.20 $$\begin{aligned} {\mathbb {E}}\left( f_{t+1|t}^{k}\left( f_{t|t-N}^{k}\right) ^{T}\right) ={\mathcal {A}}{\mathbb {E}}\left( f_{t|t-N}^{k}\left( f_{t|t-N}^{k}\right) ^{T}\right) . \end{aligned}$$

Again, identifiability of the system parameters can been shown if we show that the matrix ${\mathbb {E}}\left( f_{t|t-N}^{k}\left( f_{t|t-N}^{k}\right) ^{T}\right)$ is non-singular. This is proved as follows: For $k_{0}=np-1$ it follows that

$$\begin{aligned} f_{t|t-N}^{k_{0}}=\underbrace{{\mathbb {E}}\left( f_{t}\left( Y_{t-N,k_{0}}^{-}\right) ^{T}\right) }_{Z_{0}^{\text {g}}}\Gamma ^{ff}\left( k_{0}\right) ^{-1}Y_{t-N,k_{0}}^{-} \end{aligned}$$

since $\Gamma ^{ff}\left( k_{0}\right)$ is again positive definite. Therefore it follows that ${\mathbb {E}}\left( f_{t|t-N}^{k}\left( f_{t|t-N}^{k}\right) ^{T}\right) =Z_{0}^{\text {g}}\Gamma ^{ff}\left( k_{0}\right) ^{-1}\left( Z_{0}^{\text {g}}\right) ^{T}$ is generically non-singular since $Z_{0}^{\text {g}}$ has generically full row rank (see Koelbl 2015). Using (2.20), a consistent estimation procedure is obtained analogously to the stock case described above. The innovation covariance matrix $\Sigma _{\nu }$ can be estimated as in Koelbl et al. (2016).

## Simulations

In this section we present a simulation study comparing the accuracy of IVL with the accuracy of the XYW estimator and comparing these procedures as initial estimators for the EM algorithm. We consider the following data generating processes corresponding to the following two models:

### Example 1

Model 1 (which was also presented in Koelbl et al. (2016)) is of the form:

3.1 $$\begin{aligned} y_{t}=\begin{pmatrix}-1.2141 &{} 1.1514\\ -0.9419 &{} 0.8101 \end{pmatrix}y_{t-1}+\nu _{t}, \end{aligned}$$

and Model 2 is of the form:

3.2 $$\begin{aligned} y_{t}=\begin{pmatrix}1.5284 &{} 0.2727 &{} 1.0181\\ 1.6881 &{} -1.5235 &{} -1.1424\\ -0.6785 &{} 1.0936 &{} 1.2108 \end{pmatrix}y_{t-1}+\begin{pmatrix}-0.8089 &{} 0.4224 &{} 0.1477\\ -0.4461 &{} -0.9209 &{} -0.3154\\ -0.0496 &{} 0.6999 &{} -0.0982 \end{pmatrix}y_{t-2}+\nu _{t}. \end{aligned}$$

In both cases the innovations are standard normally distributed, i.e. $\nu _{t}\sim {\mathcal {N}}(0,I_{i})$, $i=2,3$.

The simulation study reports the mean squared errors

$$\begin{aligned} \mathrm{MSE}\left( {\hat{\theta }}\right) =\frac{1}{m}\sum _{j=1}^{m}\sum _{i=1}^{n^{2}p}\left( \theta _{i}-{\hat{\theta }}_{i}^{j}\right) ^{2} \end{aligned}$$

for the parameters $\theta =\text {vec}\left( A_{1}\right)$ and $\theta =\text {vec}\left( A_{1},A_{2}\right)$, respectively. The sample size is $T=500$ and we performed $m=10^{3}$ simulation runs. Only the case of stock variables has been considered. We put $N=2$ and $n_{s}=1$. The following estimation procedures are compared in this study: The Yule-Walker estimator obtained from high-frequency data, denoted by HF-YW. This estimator serves as an overall benchmark and therefore also the mean squared errors relative to the mean squared errors of the HF-YW estimators are presented. By MF-XYW we denote the mixed-frequency XYW estimator, by MF-IVL the mixed-frequency estimator introduced in the paper. By MF-EM-XYW we denote the mixed-frequency EM algorithm initialized with the XYW estimator and MF-EM-IVL the mixed-frequency EM algorithm initialized with the MF-IVL estimator, respectively. Table 1 summarizes the results.

**Table 1:** Absolute and relative mean squared errors of the system parameters

	Estimators	Model 1		Model 2	
		Absolute	Relative	Absolute	Relative
HF	YW	0.002	1	0.017	1
MF	XYW	0.315	133.67	0.721	43.52
	IVL	0.056	23.53	0.075	4.55
	EM-XYW	0.006	2.38	0.066	3.99
	EM-IVL	0.004	1.74	0.027	1.64

Note that for the two models MF-IVL outperforms MF-XYW as far as the overall mean squared errors are concerned. This also holds for the estimators for the individual system- as well as for the corresponding estimates of the noise parameters. When used as initial estimators, again, MF-IVL outperforms MF-XYW. In addition, the number of iterations for the EM algorithm decreases for both models when initialized with the MF-IVL instead of the MF-XYW.

## Conclusions

This paper proposes a new estimation procedure in the framework of VAR models and mixed-frequency data. The procedure is obtained by creating instrumental variables by projecting the slow variables on present and past fast ones. We show generic consistency of the system parameters for stock and flow variables. Simulations are presented to compare the properties of our procedure compared to the XYW estimator. Both procedures are less accurate when compared to the MLE, our procedure however outperforms the XYW estimator.
